# Clinical Analysis on Alteration of Thyroid Hormones in the Serum of Patients with Acute Ischemic Stroke

**DOI:** 10.4061/2010/290678

**Published:** 2010-08-09

**Authors:** Yonghua Zhang, Michael A. Meyer

**Affiliations:** Jacobs Neurological Institute, Department of Neurology, The State University of New York at Buffalo, 100 High Street, Buffalo, NY 14203, USA

## Abstract

Low T3 has been associated with increased short-term mortality in intensive care unit and long-term mortality in cardiovascular disease. The objective of this retrospective study is to investigate associations of thyroid hormone status with clinical severity and outcome in acute ischemic stroke, and whether there is association between the pituitary axis abnormality and the anterior/posterior circulation involvement. Patients with no history of thyroid abnormality who presented first ever stroke were studied. Total T3, T4, TSH levels, basic and clinical characteristics were collected and categorized. Neurological impairment was assessed using NIHSS and modified Rankin Scale. Twenty-nine patients (61%) had T3 ≤ 75 ng/dL. Low T3 group had significant higher NIHSS compared to normal T3 group. There was a significant negative correlation between T3 levels and NIHSS scores on admission. A significantly smaller percentage of patients with low T3 showed favorable neurological function improvement by both NIHSS and mRS measures compared to those with normal T3. There was no significant difference for anterior or posterior circulation involvement between low T3 and normal T3 groups. *It is suggested that low* T3 is associated with worse neurological outcome. The severity of low T3 may be a predictor of functional improvement in acute ischemic stroke.

## 1. Introduction

Neuroendocrine profile is significantly altered in acute ischemic stroke; low triiodothyronine (T3) has been associated with increased short-term mortality in intensive care unit patients and long-term mortality in patients with heart disease [1–4]. This is known as nonthyroidal illness syndrome (NTIS; or euthyroid sick syndrome or “low-T3 syndrome”). The most common hormone pattern in NTIS is a decrease in T3 level with normal levels of thyroxine (T4) and thyroid-stimulating hormone (TSH) [2, 5]. For more severe illness, a decrease in T4 level occurs while the TSH level does not show the expected pituitary thyroid axis reactivity [2, 5]. 

In acute stroke, several factors such as C-reactive protein (CRP), glucose levels on admission, fibrinogen concentration, erythrocyte sedimentation rate, and leukocyte count have been examined as prognostic factors for stroke outcome and have been found to be associated with the increased morbidity and mortality [6–8]. A reduction of serum T3 without elevation of thyroid-stimulating hormone appears to be associated with the severity of stroke and worse clinical outcome [9, 10]. Thus far, there were two studies that have been published to address the importance of monitoring for NTIS after acute stroke. Hama et al. from Japan reported malnutrition and nonthyroidal illness syndrome after stroke in 2005 [9]; another one was by Alevizaki et al. who described a low T3 levels is associated with outcome in acute stroke patients from Greece in 2007 [10]. The probable correlation between the decrease of thyroid hormones and the severity of stroke as well as the poststroke recovery needs further investigation. The objective of this pilot, retrospective study is to investigate possible associations of thyroid hormone status with clinical severity using National Institutes of Health Stroke Scale (NIHSS) and outcome in patients admitted for acute ischemic stroke, and whether there is association between the pituitary axis abnormality and the anterior or posterior circulation involvement.

## 2. Subjects and Methods

### 2.1. Study Population

Resident Research Committee of the Jacobs Neurological Institute and Institutional Review Board of University at Buffalo approved this study. All available charts of 780 patients admitted with ischemic stroke at Millard Fillmore Gates Circle hospital in Buffalo, New York in 2009 were reviewed. Inclusion criteria: (1) any patients with no history of thyroid abnormality who presented first ever stroke within 24 h from symptoms' onset; (2) patients with thyroid functions including total T3, T4, and TSH obtained in the morning following admission; (3) age 18 years and older; (4) patients with available normal thyroid function tests within one year prior to admission. Exclusion criteria: (1) age less than 18; (2) patients with thyroidal dysfunction, transient ischemic attacks, subarachnoid or intraparenchymal hemorrhage, severe liver disease (e.g., liver cirrhosis), and severe kidney disease (e.g., require artificial dialysis).

### 2.2. Data Collection and Source

Kaleida Health Infoclique electronic record system and paper charts were utilized. Head CT, MRI, MRA official reports, physician dictations, discharge and/or transfer summaries, and laboratory works were carefully reviewed. Thyroid function was evaluated by measuring serum total T3, T4, and TSH; T3, T4 were measured by RIA (radioimmunoassay) and TSH by IRMA (immunoradiometric assay) at Kaleida Health System. Normal range in our laboratory for T3 is 75–180 ng/dL; normal range for T4 is 5–11.5 mcg/dl; normal range for TSH is 0.4–5 *μ*U/mL. Total T3, T4, and TSH levels obtained in the morning following admission were collected. 

Basic and clinical characteristics including demographic data such as sex, age, cigarette smoking, concurrent illness, medications (in the present study only ASA, plavix, or coumadin were taken into account, these were all for patients' coronary artery diseases), and whether the stroke involve the anterior or posterior circulation from brain imaging were collected and categorized. We divided all patients into 2 groups with one low-T3 group and other normal-T3 group based on first thyroid functions on day 1 after admission.

The severity distribution of NIHSS scores on admission was divided into 3 categories, mild: NIHSS < 8; moderate: NIHSS 8–14; severe: ≥14. Neurological impairment and improvement were assessed using NIHSS, together with modified Rankin Scale (mRS) [11, 12] from follow-up clinic visits. NIHSS scores were repeated on patients' first outpatient clinic follow-up; ΔNIHSS (improvement: a decrease of NIHSS score) was then calculated from patient's admission NIHSS to assess functional improvement. Favorable outcome is defined as ΔNIHSS ≥ 2 and mRS ≤ 1 [13]. The first clinic follow-up usually occurs 2–4 weeks after discharge from hospital.

### 2.3. Statistical Analysis

Statistical analysis was performed to compare patients with low T3 and normal T3 values. Continuous data are presented as mean (standard deviation), categorical data as percentages. Unpaired student *t*-test (for continuous variables), chi-square (for categorical variables), and correlative analysis will be used to determine if low T3 levels are associated with acute ischemic stroke severity. Differences were considered significant at *P* < .05. The Statistical Package for Social Sciences (SPSS inc., version 16.0 for Windows) was used.

## 3. Results

Forty-seven patients with available laboratory data and relatively complete neurological impairment documentations met all inclusion criteria, 28 men, 19 women. Mean age was 67.4 ± 12.1. Twenty-nine patients (61%) had T3 ≤ 75 ng/dL and 18 patients had normal T3 values. The characteristics of patients with low T3 and normal T3 are shown in Table 1. Patients with low T3 had significantly more severe neurological impairment at presentation (*P* < .05), higher mean glucose levels on admission (*P* < .05). Low-T3 group had significant higher NIHSS scores compared to normal-T3 group (*P* < .05). Patients' distribution pattern of NIHSS scores on admission showed that a much higher portion of patients belonged to moderate-to-severe category (NIHSS: 8–14 or ≥14) for low-T3 group while majority of patients fell into mild category for normal-T3 group (NIHSS < 8). There was a trend of decreased T4 levels noted but did not yield a significant difference when compared to normal-T3 group; but TSH levels showed no difference between low-T3 and normal-T3 groups. In terms of cerebral blood supply territory affected by stroke, there was no significant difference for anterior or posterior circulation involvement between low-T3 and normal-T3 groups (*P* > .05) (see Table 1).

**Figure 1:**
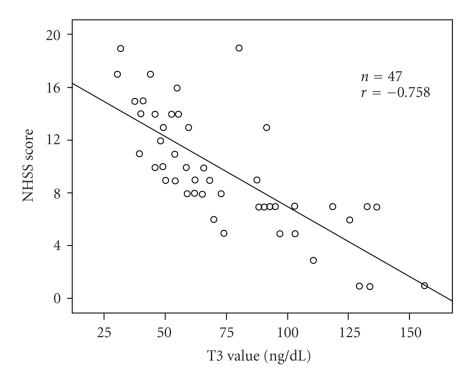
Scatter plot graph shows the correlation between T3 values and National Institutes of Health Stroke Scale (NIHSS) scores on admission. Each circle represents a patient. *r* ^2^ = 0.575, *P* < .001, 95% Confidence Interval 0.40 to 0.75.

From Figure 1, there was a significant negative correlation between T3 levels and NIHSS scores among all patients (*n* = 47, *r* = −0.758, *r* ^2^ = 0.575, *P* < .001, 95% Confidence Interval 0.40 to 0.75). This implies, after acute stroke, that the lower T3 values are, the worse neurological impairment will be.

During patients' first outpatient clinic follow-up after stroke, NIHSS scores were repeated along with mRS scores; ΔNIHSS was calculated from patient's admission NIHSS scores to assess function improvement. We found that a smaller percentage of patients with low T3 showed favorable neurological function improvement by both NIHSS measure (27.6% versus 66.7%, *P* < .05) and mRS measure (31.0% versus 55.6%, *P* < .05) compared to those with normal T3; in addition, median HIHSS and mRS scores tended to be worse in low-T3 group when compared to normal-T3 group on first clinic follow-up visit (*P* < .05; See Table 2).

## 4. Discussion

The major finding of this pilot, retrospective study showed the association of T3 levels with short-term outcome after the event of acute ischemic stroke. Our finding from the present study has not been previously reported in the United States using NIHSS measure. The most common abnormality is low T3 among all four types of NTIS in hospitalized especially those in intensive care units [14, 15]. Low T3 has been reported in intracerebral hemorrhage from either trauma or hemorrhagic infarct [16, 17]. It has been generally accepted that low T3 accompanying severe illness is considered as an adoptive response to stress to spare energy [1–4, 14, 18]. Whether or not to treat with thyroxin replacement is controversial [2, 5, 19–22], while true hypothyroidism has been interestingly reported, that a preexisting condition may actually be protective in acute stroke [23, 24]. Like in other systemic severe illness, a reduction of serum T3 without elevation of thyroid-stimulating hormone appears to be associated with the severity of stroke and worse clinical outcome [9, 10, 25]. Possible mechanisms include, but not limited to, the following: (1) peripheral thyroid hormone metabolism changes due to alterations in activity of the enzymes responsible for peripheral conversion of T4 to T3 [26, 27]; (2) proinflammatory cytokine action involvement; (3) a disturbed shift in the distribution of thyroid hormones or an alteration in binding proteins [28, 29]; (4) excessive glucocortcoids releases in severe illness which produce inhibition of activities from hypothalamic-pituitary-thyroid axis and the conversion of T4 to T3 [30]. Our study showed that patients with low T3 were slightly but not significantly older than those with normal T3; but Alevizaki et al.'s study showed significant older age in low-T3 group [10]. This may have represented that elderly patients usually had a low baseline of thyroid function and tended not to fully compensate when such event occurs. Patients from variable age group would have helped in the present study.

In the present study, low T3 appeared to be associated with stroke severity and short-term outcome; furthermore, there is a significant negative correlation detected between T3 levels and NIHSS scores on admission among all patients. The latter is a novel finding which has not been reported elsewhere, it indicates that a worse neurological impairment is related to the degree of a decrease in T3 level, thus T3 levels monitoring could potentially serve as an easy, quick, and feasible prognostic parameter for clinicians in the future if confirmed by further studies [18]. Our finding is consistent with Alevizaki et al. who stated that low-T3 is a possible independent predictor for stroke outcome [10]. As in several studies performed in intensive care units, acute stroke patients with low T3 or the combination of T3 and T4 appeared to be associated with worse prognosis [1–4, 31]. Also, interestingly the alterations of T3 levels do not appear to be related to the region (anterior versus posterior circulation) affected by stroke. This suggests that the alteration is more related to a disturbance of thyroid hormone metabolism rather than a blood supply abnormality induced structural disturbance of the hypothalamic-pituitary-thyroid axis.

Limitations in the present study include selection biases, small sample size, single baseline measurement of thyroid function, lack of long-term follow-up, and interactions from any drugs known to affect thyroid functions which should all be taken into account in the future study. Among the above items, the confounding effects from a few selection biases such as whether patients had available laboratory results for thyroid function tests and whether they presented to the hospital deserve readers' attention while reviewing or using these data. Following studies are needed in the future: (1) to monitor repeatedly TSH, T3, and T4 levels at specific times relative to onset of stoke (i.e., at onset, 1st day, 1st week, 1 month, 3 months); (2) to perform an adjusted analysis such as logistic regression and Mantel-Haenszel test to exam if T3 is indeed an independent predictor along with other factors and whether the association between low T3 syndrome and worse neurologic impairment is truly significant. Since there is still no conclusive answer as to treatment of low T3 in the context of acute stroke, it would be also conceivable to design a study on influential effect by providing proper thyroid hormone replacement. Some studies reported advantage of brain “being preconditioned” with hypothyroidism when a stroke occurs [10, 23, 24], this result has led to an extensive discussion on the issue of correction of low T3 levels after stroke [19, 32]; whereas one animal study showed a neuroprotective effects defined by reduction of infarct size and improvement of neurological deficit with administration of T3 [33].

 In conclusion, NTIS is commonly seen after acute ischemic stroke, and low T3 is associated with worse neurological outcome. The severity of low T3 syndrome may be a useful predictor of functional improvement in patients with acute ischemic stroke. The study suggests that thyroid hormone monitoring poststroke may be considered if the present data are further confirmed by future prospective studies.

## Figures and Tables

**Table 1:** Characteristics of acute ischemic stroke patients with low T3 and normal T3 values.

	Low T3 group	Normal T3 group	*P*-value
	(T3 ≤ 75 ng/dL)	(T3 > 75 ng/dL)	
	*n* = 29	*n* = 18	
*Clinical variables*			
Age (years)	68.1 (±12.7)	66.8 (±11.5)	NS
Sex (% male)	18 (62.1%)	11 (61.1%)	NS
Smoking	9 (31.0%)	6 (33.3%)	NS
Hypercholesterolemia	8 (27.6%)	5 (27.8%)	NS
Coronary artery disease	5 (17.2%)	3 (16.7%)	NS
Diabetes mellitus	9 (31.0%)	6 (33.3%)	NS
Atrial fibrillation	6 (20.1%)	4 (22.2%)	NS
History of hypertension	19 (65.5%)	11 (61.1%)	NS
BP on admission			
Systolic BP (mmHg)	156.2 (±27.9)	150.2 (±29.5)	NS
Diastolic BP (mmHg)	89.7 (±11.4)	87.8 (±14.2)	NS
Baseline NIHSS scores	11.5 (±3.5)	6.6 (±4.3)	<.05
Distribution of NIHSS scores			
Mild (<8)	2 (6.9%)	15 (83.3%)	<.01
Moderate (8–14)	17 (58.6%)	2 (11.1%)	<.01
Severe (≥14)	10 (34.5%)	1 (5.6%)	<.01

*Laboratory variables*			
Anterior carotid artery territory	11 (37.9%)	7 (38.9%)	NS
Posterior/basilar artery territory	6 (20.1%)	4 (22.2%)	NS
Thyroid function tests			
TSH (*μ*U/mL)	1.24 (±1.19)	1.30 (±1.21)	NS
T3 (ng/dL)	53.1 (±11.7)	109.5 (±21.6)	<.01
T4 (ng/dL)	8.5 (±3.04)	9.2 (±2.6)	NS
Initial WBC count	7.8 (±3.9)	7.3 (±2.5)	NS
Initial hemoglobin	9.4 (±1.4)	9.1 (±1.8)	NS
Glucose on admission (mmol/L)	135.6 (±56.8)	128.9 (±55.4)	<.05

*Medications*			
Antiplatelets/Anticoagulants	14 (48.3%)	9 (50%)	NS

Numbers for nominal data indicate percentages in parentheses and for continuous data indicate mean ± standard deviation. Statistical significance (*P* < .05). NS: no statistical significance; BP: blood pressure; NIHSS: National Institutes of Health Stroke Scale; Baseline NIHSS scores: the initial scores on admission; TSH: thyroid-stimulating hormone; T3: triiodothyronine; T4: thyroxine; Antiplatelets/Anticoagulants: ASA or Plavix or coumadin.

**Table 2:** Stroke outcome measured by National Institutes of Health Stroke Scale (NIHSS) and modified Rankin Scale (mRS).

	Low T3 group	Normal T3 group
	(T3 ≤ 75 ng/dL)	(T3 > 75 ng/dL)
	*n* = 29	*n* = 18
*The 1st day*		
Median value of NIHSS	11 (5~19)	6 (2~14)*

*The 1st clinic follow-up*		
Median value of NIHSS	9 (2~17)	4 (0~9)*
Patients with any improvement (ΔNIHSS ≥ 2)	8 (27.6%)	12 (66.7%)*
Median value of mRS	3 (1~5)	1 (0~3)*
Patients with favorable outcome (mRS ≤ 1)	9 (31.0%)	10 (55.6%)*

Numbers in parentheses for nominal data indicate percentages and for continuous data indicate range of actual scores. *P < .05 is compared to low T3 group. The 1st day: on admission; The 1st clinic follow-up: outpatient follow-up usually occurred 2–4 weeks after discharge; ΔNIHSS ≥ 2: improvement when scores decreased ≥2 compared to baseline.
